# Critical Review on Molecular Mechanisms for Genistein’s Beneficial Effects on Health Through Oxidative Stress Reduction

**DOI:** 10.3390/antiox14080904

**Published:** 2025-07-24

**Authors:** Ke Zhang, Jingwen Wang, Baojun Xu

**Affiliations:** Food Science and Technology Program, Department of Life Sciences, Beijing Normal-Hong Kong Baptist University, Zhuhai 519087, China; r130013040@mail.uic.edu.cn (K.Z.); wangjingwen@uic.edu.cn (J.W.)

**Keywords:** genistein, oxidative stress, signaling pathway, antioxidant, anti-inflammatory

## Abstract

Oxidative stress directly or indirectly contributes to the development and progression of various diseases; therefore, regulating oxidative stress is a promising strategy for preventing or treating these conditions. The unique substances in soybeans, soy isoflavones, notably genistein, which have a strong antioxidant capacity, are considered to regulate various signaling pathways, alleviate oxidative stress, and improve gut microbiota imbalance as well as mitochondrial dysfunction. In this literature review, we summarize the latest research on genistein, providing evidence of its development and application as a potential drug for preventing and treating five selected diseases (Parkinson’s disease, Alzheimer’s disease, diabetes mellitus, cardiovascular disease, and cancers). The literature was searched using keywords that include tripartite combinations of genistein and oxidative stress, along with each of the five selected diseases, from PubMed, Science Direct, and Google Scholar between 2014 and 2024. According to current in vitro, in vivo, and clinical trials, we comprehensively discuss the therapeutic dose used to target various disease entities to achieve optimal efficacy and meet safety requirements. Moreover, considering the poor water solubility and limited bioavailability of genistein, strategies for improving its therapeutic efficacy, such as combining it with exercise, existing medications, and advanced technologies, as well as applying nanotechnology, were assessed. Therefore, this review aims to provide robust evidence for the development and application of genistein as a potential therapeutic agent or functional food for preventing and treating these diseases.

## 1. Introduction

**Figure 1:**
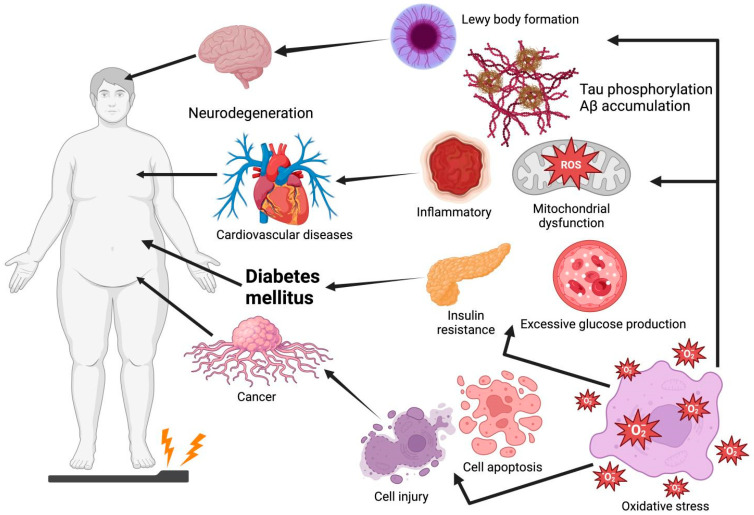
Oxidative stress leads to Lewy body formation, tau phosphorylation, Aβ accumulation, insulin resistance, excessive glucose production, cell injury, and cell apoptosis, which are common causes of Parkinson’s disease, Alzheimer’s disease, cardiovascular disease, diabetes mellitus, and cancer. Graphical elements were created using BioRender (https://www.biorender.com).

Oxidative stress is an imbalance between the decline in antioxidant mechanisms and the growth of free radicals, such as reactive nitrogen species (RNS) and reactive oxygen species (ROS), leading to the overproduction of oxidative free radicals that damage lipids, proteins, and DNA [1]. Among these, ROS, a highly reactive metabolic byproduct and biomarker of oxidative stress, play a dual role in cell function and reduction–oxidation (redox) homeostasis. At low to intermediate levels, ROS play a positive role in cellular functions (e.g., proliferation, differentiation, migration, apoptosis, and necrosis). However, excessive ROS production leads to oxidative stress and chronic diseases owing to factors such as mitochondrial deficiency and nutritional stress [2,3]. Thus, oxidative stress is considered a causative factor for the onset of numerous diseases such as Parkinson’s disease (PD), Alzheimer’s disease (AD), diabetes mellitus (DM), cardiovascular diseases (CVDs), and cancer (Figure 1). For instance, plaque aggregation and the high phosphorylation of tau, induced by oxidative stress, are two of the main causes of AD [4]. Moreover, oxidative stress increases the risk of insulin resistance (IR) by impairing insulin signaling transduction [5]. Thus, future drug interventions should maximize the amelioration or prevention of diseases by inhibiting oxidative damage, especially using non-toxic and absorbable natural products.

Genistein is a flavonoid commonly found in soy products. It is a phytoestrogen that can combine with estrogen receptors in the body to mimic estrogen and benefit lipid metabolism in postmenopausal women. It has shown excellent anti-aging, anti-inflammatory, and anti-apoptotic abilities, possibly protecting targeting cells and activating critical signaling pathways such as nuclear factor erythroid 2-related factor 2 (Nrf2) or phosphatidylinositol 3-kinase/protein kinase B (PI3K/Akt) against ROS damage. For example, genistein increased the expression of superoxide dismutase (SOD), catalase (CAT), glutathione peroxidase (GPx), and other related enzymes to regulate ROS levels and reduce the incidence of diseases [6]. Numerous preclinical studies have suggested that genistein increases SOD protein expression by upregulating the Nrf2 pathway and the PI3K/Akt pathway as well as downregulating the mitogen-activated protein kinase (MAPK) pathway, especially jun N-terminal kinase (JNK) and p38 MAPK, which decreases the prevalence of aging, AD, PD, CVD, and cancer (e.g., bladder cancer, liver cancer, and breast cancer) induced by ROS [7]. Thus, genistein can have beneficial health effects by increasing the expression of some antioxidant enzymes to reduce the adverse effects caused by unbalanced oxidative stress.

However, the molecular mechanism of genistein treatment has not been thoroughly investigated, and its efficacy in alleviating diseases caused by oxidative stress remains to be determined. Thus, this review aims to summarize the molecular mechanisms by which genistein reduces diseases caused by oxidative stress and further demonstrate the curative effect of genistein on such diseases, thereby assisting in substantiating some hypotheses for the positive therapeutic role of genistein and serving as a reference for selecting different dosage levels in practical research experiments.

Also of note, this article, in the form of a review, serves as a medium to summarize the positive therapeutic effects of genistein on five common diseases that have become increasingly prevalent in recent years. It provides readers interested in the antioxidant effects of genistein with a systematic understanding of its potential in disease treatment through the regulation of oxidative stress, a prominent advantage of the review format. All comments in this review are supported by a substantial body of experimental data, including preclinical experience and actual clinical trials, which highlight the practical potential of plant extracts in therapeutic applications. At the same time, by covering various treatment approaches and dosages for each disease, the review enables readers to compare the relative costs and effects of each approach horizontally. Moreover, given that scientists have expressed reservations about genistein’s ability to treat or prevent these diseases due to its limited bioavailability, various strategies for enhancing its therapeutic efficacy have been explored in this article, including its synergistic effects with exercise and existing medications, as well as the research and development of genistein-loaded nanoparticles. This also further provides readers with a new understanding of both the challenges and the latest advancements in enhancing the therapeutic efficacy of genistein.

## 2. Methodology

This review was conducted by investigating the most relevant literature in the scientific databases PubMed (https://pubmed.ncbi.nlm.nih.gov, assessed on 1 January 2025), Science Direct (https://www.sciencedirect.com, assessed on 3 January 2025), and Google Scholar (https://scholar.google.com, assessed on 5 January 2025). Relevant articles were published from 2014 to 2024, with most published in the past five years. The keywords included tripartite combinations of genistein and oxidative stress with the five selected diseases (PD, AD, DM, CVD, and cancer). Those that did not cover the research topic of interest or that lacked appropriate design descriptions were excluded from this review. Therefore, we have summarized the molecular mechanisms involved in the specific palliative or therapeutic effects of genistein on five common diseases, including the in vivo and in vitro pharmacological effects of genistein and the most recent clinical trials.

## 3. The Characteristics of Genistein and Its Therapeutic Effect Through Inhibiting Oxidative Stress

### 3.1. The Chemical Structure of Genistein

Genistein, a 4,5,7-trihydroxy isoflavone, is recognized and used as a phytoestrogen owing to its chemical structure resembling that of mammalian estrogen. This similarity enables genistein to play beneficial roles, providing anxiolytic, neuroprotective, cardioprotective, anticarcinogenic, anti-inflammatory, and antioxidant effects [8,9,10].

**Figure 2:**
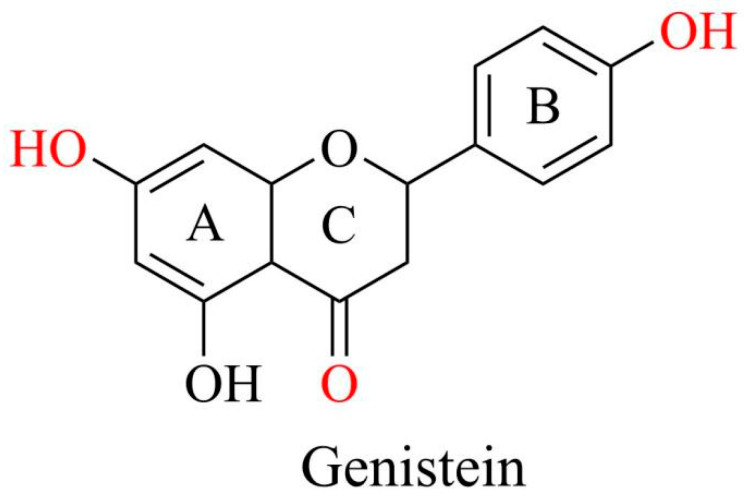
Chemical structures of genistein. Graphical elements were created using Chemdraw (https://www.chemdraw.com.cn).

The molecular formula of genistein is C_15_H_10_O_5_ with a molecular weight of 270.241 g/mol. It comprises 15 carbons arranged in two aromatic rings (A and B) and a central pyran ring (ring C). A distinctive feature is the double bond between the second and third positions, which constitutes the fundamental carbon skeleton of genistein [8]. The key functional groups of genistein are the 7-position hydroxyl group of the A ring, 4-position hydroxyl group of the B ring, and 4-position ketone group of the C ring, which are mainly responsible for the biological activity of genistein. The four and seven hydroxyl groups are directly involved in antioxidant activity, binding to estrogen receptors, and regulating key signaling pathways such as PI3K/Akt. The ketone group provides molecular polarity and facilitates the formation of intermolecular hydrogen bonds (Figure 2). Additionally, the three hydroxyl groups of genistein enhance estrogenic activity, increasing its substantial capacity to interact with estrogen receptors and regulate various disorders [11].

### 3.2. The Therapeutic Effect of Genistein Through Inhibiting Oxidative Stress 

As for its antioxidant capacity, genistein is a dose-dependent substance with a dual effect against oxidative stress. At relatively low levels, genistein enhances antioxidant enzymes to protect the human body from the destructive damage caused by oxidative stress [12]. When the concentration of genistein is high, although it can also reduce the likelihood of the further progression of cerebral infarction and liver damage in women, it acts as a pro-oxidative stress factor and exacerbates ROS production in the human body [13,14].

On the one hand, at low concentrations, genistein, as a critical upstream stimulatory factor, positively affected many phase II detoxification and antioxidant enzymes through nuclear factor kappa B (NF-κB) to regulate many diseases [15]. Moreover, genistein inhibited the development of neonatal hypoxic-ischemic brain damage (HIBD) by upregulating the NF-κB pathway and altering interleukin-6 (IL-6), tumor necrosis factor-α (TNF-α), and interleukin-1β (IL-1β) [16]. Genistein upregulated the NF-κB pathway and *Nrf2* expression, ultimately inhibiting oxidative stress. Then, it effectively improves oxidative stress and neuroinflammation caused by cerebral ischemia due to NF-κB restraint and antioxidants in the Nrf2 pathway [17]. Similarly, in hypoxic-ischemic encephalopathy therapy, genistein upregulated the erythroid 2-related factor transcription factor/hemoxygenase 1 (Nrf2/HO-1) pathway and inhibited the NF-κB signaling pathway. Genistein diminished brain infarct size and neuronal apoptosis while enhancing neuroprognosis and brain atrophy recovery, leading to the attenuation of oxidative stress and neuroinflammation induced by hypoxic-ischemic brain injury [16]. After genistein treatment, the viability of HIBD cells after oxygen–glucose deprivation/reoxygenation injury increased, whereas neuronal injury and cell apoptosis levels decreased. Notably, no toxic effects can be detected after genistein entered the blood–brain barrier, indicating its potential in brain damage therapy [16].

In contrast, high concentrations of genistein are pro-oxidative stress factors that stimulate ROS production and damage the human body. ROS alter the conformation and detachment of Kelch-like ECH-associated protein 1 (Keap1), along with the intensified ubiquitination and proteolysis of Nrf2 in the cytoplasm, enhancing the interaction between Nrf2 and Keap1 [15,18]. When Nrf2 was translocated into the nucleus and bound to the musculoaponeurotic fibrosarcoma oncogene homolog protein, genistein was attached to antioxidant response elements and activated antioxidant gene expression [19]. Consequently, genistein triggered HO-1, SOD, and CAT activities through Nrf2 release and increased antioxidant response element expression, leading to severe oxidative stress [20]. High concentrations of genistein may lead to hormonal imbalances and endocrine disruption [21,22]. As a phytoestrogen, genistein inhibited natural estrogen activity by competing with endogenous estrogen for receptor binding. In addition, excessive genistein intake potentially increased the risk of hormone-sensitive conditions or the prevalence of cancer, such as breast cancer or endometrial hyperplasia, in susceptible individuals [23].

## 4. Therapeutic Application of Genistein in Common Oxidative Stress-Induced Diseases

**Figure 3:**
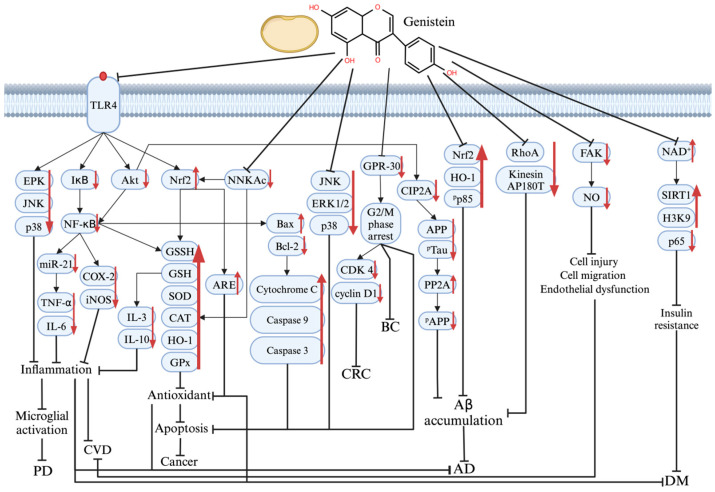
The mechanism of genistein’s therapeutic effect on various protein and signaling pathways in PD, AD, DM, CVD, and cancer pathogenesis. Graphical elements were created using BioRender (https://www.biorender.com). ↑ means increase; ↓ means decrease; ┤ means repressed.

The multiple molecular mechanisms underlying the therapeutic effects of genistein on PD, AD, DM, CVD, and cancer are shown in Figure 3. 

### 4.1. Parkinson’s Disease (PD)

PD is a progressive disorder of the nervous system that primarily affects individuals over the age of 60 years. PD is characterized by the unbinding of inflammatory cytokines (e.g., IL-6, TNF-α, and IL-1β) due to microglial activation by the formation of Lewy bodies as well as lipopolysaccharide and dopaminergic neuron loss in the dorsomedial cluster and substantia nigra (SN). Lewy bodies, which contain overexpressed α-synuclein (αS), are the neuropathological hallmark of PD and critical targets for treatment strategies [24].

Genistein, a polyphenol, has the potential to alleviate PD symptoms with few side effects [25]. Anti-inflammatory effects, antioxidant activity, and anti-apoptotic properties help protect dopaminergic neurons and slow the progression of PD. Genistein inhibited MAPK and IκB signaling pathways and reduced cyclooxygenase-2 (COX-2) and inducible nitric oxide synthase (iNOS) expression in the SN of rats through G protein-coupled estrogen receptor and insulin-like growth factor 1 receptor signaling pathway regulation [26]. The NF-κB signaling pathway was restricted, while the 5′ AMP-activated protein kinase (AMPK) signaling pathway was activated by genistein. Therefore, the inflammatory response in the human synovial membrane of TNF-α-induced MH7A cells was successfully suppressed. In ovariectomized rats induced by amyloid-beta (Aβ), genistein downregulated the expression of MAPK, Toll-like receptor 4 (TLR4), and NF-κB in microglia, offering neuroprotection against inflammation, reducing microglial activation, protecting SN dopaminergic neurons, and lowering the phosphorylation levels of extracellular signal-regulated kinase (ERK), p38, JNK, and IκB [26].

Genistein has a good antioxidant effect, protecting neurons from oxidative damage. An in vitro study investigated the anti-Parkinson effects of genistein (20 μM) on cell viability, apoptosis, and oxidative stress in human SH-SY5Y cells treated with rotenone. The results showed that the genistein intervention increased cell viability, reduced apoptosis, and attenuated the oxidative stress imbalance, primarily through the activation of estrogen receptors and the nuclear factor erythroid 2-related factor 2 signaling pathway [27]. Similarly, genistein treatment at 40 μM in transgenic *Drosophila* was reported to enhance antioxidant capacity, thereby further protecting neurons. Genistein reduced lipid peroxidation by 2.5 times compared to untreated *Drosophila* PD at this concentration. Genistein increased the glutathione (GSH) levels, minimized oxidative stress, and protected neurons by balancing ROS production and oxidative conditions through lower monoamine oxidase activity [28]. However, human αS and Lewy bodies were present in both treated and untreated *Drosophila*, indicating that genistein’s therapeutic effect on PD was unable to stem αS expression or Lewy body formation and only enhanced antioxidant ability [24]. Moreover, genistein inhibited the formation of some ROS producers (dopamine, 3,4-Dihydroxyphenylacetic acid, and homovanillic acid ) in the treated group, demonstrating its inhibitory effect on oxidative stress [26].

Genistein also controls caspase-8 and caspase-3 to mitigate apoptosis in PD pathological features. Genistein downregulated death receptors, such as tumor necrosis factor receptor superfamily member 6 (*FAS* and TNF, limiting caspase-8 recruitment to the death-inducing signaling complex. It also decreased the levels of the pro-inflammatory cytokine TNF-α, which had a positive relationship with caspase-8 in terms of its anti-inflammatory properties. Genistein prevented oxidative damage to mitochondrial DNA, proteins, and lipids by stabilizing mitochondrial function and reducing ROS levels, thereby avoiding further apoptosis [29]. Moreover, genistein upregulated SOD, GPx, and Bax (Bcl-2-associated X protein) and decreased β-cell lymphoma 2 (Bcl-2) and Bid levels, hindering the growth of both caspase-8 and caspase-3. As mentioned in the review by Singh et al., genistein alleviated the cytotoxic effects of 6-hydroxydopamine (6-OHDA) in the PC12 cell model by inhibiting the activation of caspase-8 and caspase-3 proteins [25].

In conclusion, these in vivo and in vitro experiments elucidated the molecular mechanism of genistein in PD treatment, highlighting its role in attenuating inflammatory responses and oxidative damage, as well as modulating caspase-8 and caspase-3 expression. Notably, although the current experiments found that genistein does not reduce αS expression, future studies should explore its potential in neural stem cell therapy or as an adjuvant therapy to enhance therapeutic strategies for PD by targeting αS reduction in combination with other drugs.

### 4.2. Alzheimer’s Disease (AD)

AD is a common neurodegenerative disorder that is characterized by progressive memory loss and cognitive decline. In the early stages of AD, microglial activation around Aβ deposition sites increases the levels of pro-inflammatory cytokines. ROS cause lysosomal dysfunction and neuroinflammation, contributing to Aβ accumulation and tau phosphorylation, thereby advancing neurodegeneration [30,31,32].

Genistein, a cell-permeable protein tyrosine kinase inhibitor, regulates various intracellular signaling pathways by the auto-phosphorylation of the epidermal growth factor receptor kinase. It reduced the formation of TNF-α and TLR4, which were typical pro-inflammatory molecules, improving memory and alleviating astrogliosis in SH-SY5Y cell models with AD [33]. Duan et al. have summarized in their literature review that the anti-inflammatory effects of genistein may be associated with the suppression of the TLR4-mediated NF-κB signaling pathway [34].

Genistein has been reported to increase antioxidant effects by blocking Aβ toxicity in cell and animal models. For instance, in the Aβ_25–35_ rat primary hippocampal neuron model, genistein significantly increased cell viability while suppressing ROS production, MDA levels, LDH levels, and apoptosis. Further results suggested that α7nAChR signaling-mediated PI3K/Akt and Nrf2/Keap1 pathways are critical for the neuroprotective effect of genistein [35]. Genistein significantly reduced amyloid plaques in the anterior cingulate gyrus [36]. Genistein reduced amyloid precursor protein (APP) secretion by inhibiting β-site APP-cleaving enzyme 1 and blocking platelet-derived growth factor stimulation [37]. The anti-neurotoxicity ability of genistein, mediated through the Nrf2/HO-1/PI3K pathway, was demonstrated in an Aβ_25–35_-treated SH-SY5Y cell model, showing the inhibitory effect of genistein on Akt and tau phosphorylation, thereby strengthening resistance to Aβ-induced toxicity [34]. Meanwhile, 50 μM genistein upregulated HO-1 expression and strengthened cells’ defense in cases of oxidative stress injury, increasing the survival rate of SH-SY5Y cells [33]. It also reduced Aβ_42_-induced neurotoxicity and accumulation by inhibiting kinesin AP180T and Ras homolog family member A (RhoA) [6]. High concentrations of genistein regulate tau phosphorylation and Aβ_40_ and Aβ_42_ levels in the cortex and hippocampus, reducing the abnormal accumulation of harmful proteins in key brain regions and thereby preventing AD development [38].

Notably, alterations in the composition of gut microbiota are highly associated with oxidative stress imbalance, neuroinflammation, and ongoing neuronal loss, causing the onset and progression of brain disorders such as AD [39]. As mentioned in our previous literature review, the consumption of soy isoflavones has been shown to improve gut homeostasis [40]. It increases the population of *Bifidobacteria*, *Eubacteria*, and short-chain fatty acid-producing *Lachnospira*, supporting longevity and improving cognitive behaviors, which may serve as an alternative for AD treatment. Moreover, S-equol (a gut microbiome-derived metabolite of genistein with antioxidant properties) is reported to decrease white matter lesions in the aging brain and lead to the amelioration of AD symptoms due to its anti-amyloid ability [40].

In summary, genistein reduces inflammation and Aβ toxicity to slow down the further development of AD pathology with limited side effects. Viña et al. observed that long-term continuous treatment with genistein (exceeding one year) significantly reduced Aβ accumulation, thereby delaying the onset of AD in patients with prodromal AD. This conclusion was based on the increased results from 18F-flutemetamol in the control group, whereas no significant changes were detected in the genistein-treated group [36]. However, further studies are required to determine the precise duration of genistein treatment required for AD management. At the same time, shortening the duration of genistein treatment in AD and improving its bioavailability are still worth considering in future development.

### 4.3. Diabetes Mellitus (DM)

DM is a prevalent disease among older people, primarily caused by methylglyoxal (MG) exposure, IR, and mitochondrial dysfunction. Oxidative stress, inflammation, abnormal blood sugar levels, and obesity increase the risk of DM [41]. Genistein is a phytoestrogen that controls insulin sensitivity and glucose homeostasis during DM treatment. It regulates β-cells and promotes efficient glucose regulation by elevating cyclic adenosine monophosphate and protein kinase A [42,43,44].

Genistein regulates nicotinamide adenine dinucleotide, oxidized form (NAD^+^) to prevent IR [45]. In skeletal muscle, the HOMA-IR results showed that genistein increased NAD^+^ levels, thereby stimulating insulin sensitivity [46,47]. In the C/EBPβ-expressing 3T3-L1 adipocyte model, genistein increased NAMPT expression to upregulate PHB1 stability. NAD^+^ levels then increased, alleviating metabolic dysfunction and decreasing IR [47,48,49].

Genistein regulates the DM process by mitigating oxidative damage. When *EA. HY926* cells were exposed to MG, the ROS level of the genistein-treated group remained stable, while that of the untreated group showed a significant increase. These results reflected the inhibitory effect of genistein on oxidative damage. Simultaneously, the reduction of Nrf2 translocation in genistein-treated cells proved the mitigation of oxidative stress, which was caused by MG [50]. In addition, genistein reduced MAPK phosphorylation and apoptosis, as well as mitigated endothelial cell oxidative damage [51]. It also reduced chronic inflammation by suppressing NF-κB, IL-1β, and TNF-α and mitigated oxidative stress in pancreatic cells, thereby preserving the function of β-cells [41].

Emerging studies have reported that individuals with T2DM exhibit reduced levels of *Bifidobacterium*, *Firmicutes*, and *Akkermansia* genera in their gut microbiota [52,53]. This gut microbiota dysbiosis causes chronic inflammation and oxidative stress, as well as disorders in glycolipid metabolism and insulin resistance, which are key factors in the pathogenesis of T2DM and contribute to its deterioration. Thus, regulating gut microbiota is considered a promising alternative for protecting against metabolic diseases. According to a recent randomized controlled trial, 2 months of dietary supplementation with genistein effectively increases the expression of fatty acid oxidation-related genes, alters the gut microbiota, and attenuates insulin resistance [45]. Moreover, in a study analyzing causation in genistein interventions, gut microbiota changes, glucose metabolism, and adipose tissue browning, it was found that the genistein treatment not only improved the diversity of gut microbiota, but also attenuated impaired glucose metabolism in mice treated with a high-fat diet, suggesting that healthy gut homeostasis is essential for metabolic health [54]. Similarly, genistein has been reported to significantly attenuate glucose and lipid metabolism dysfunction, inflammation, and insulin resistance by enhancing the abundances of *Bacteroides* and *Prevotella* and reducing those of *Helicobacter* and *Ruminococcus* in T2DM mice [55]. Thus, the alteration in gut microbiota composition and abundance induced by genistein intervention is tightly implicated in DM prevention and treatment.

In summary, genistein hinders DM through IR regulation and the prevention of oxidative damage via various signaling pathways. These do provide a compelling option for the development of novel plant-based functional drugs for DM treatment. However, in animal studies, there was no significant difference in the effect of genistein interventions between 12 and 24 weeks, suggesting that the optimal duration of genistein treatment still needs to be determined through clinical trials [42]. Meanwhile, it is worth considering whether genistein could be combined with existing antidiabetic drugs to optimize therapeutic strategies.

### 4.4. Cardiovascular Disease (CVD)

CVD has a close relationship with oxidative stress damage, leading to most causes of death worldwide, which is much greater than the cumulative sum of cancer and chronic lung disease [56]. Smoking, a high-salt diet, and oxidative stress generate inflammation, spawning endothelial dysfunction, and eventually trigger vascular lesions, vascular remodeling, thrombosis, and CVD. Inflammatory and cardiovascular system disorders are the two main factors that result in CVD damage in humans.

Genistein reduces inflammation, cell invasion, and migration by regulating the key pathways related to oxidative stress. Genistein prevents inflammation and enlargement in vascular endothelial cells (VECs) through miRNA control. VEC damage, which releases various inflammatory factors and chemokines, causes the local accumulation of lipid responses in the vascular intima. In an LPS-induced C57BL/6 mouse model, genistein downregulated the miR-21/NF-κB p65 pathway, similar to an miR-21 inhibitor, to block the growth of typical anti-inflammatory factors (TNF-α, IL-6, and iNOS), limiting inflammation in VECs [57]. In a C57BL/6J male mouse model, genistein worked as a CB1 antagonist and CB2 agonist to neutralize Δ^9^-tetrahydrocannabinol (THC), which was the psychoactive component of cannabis, thus repairing damage to the cardiovascular system. Δ^9^-THC not only caused VEC inflammation, but also aggravated endothelial dysfunction. Simultaneously, based on the decrease in NF-κB phosphorylation, genistein decreased IL-3, IL-6, and IL-10 expressions while increasing SOD and GSH expression in mouse serum, reversing the Δ^9^-THC-induced effect with little damage to the cardiac structure or function of the heart [58]. In the HU-EST cell model, genistein downregulated the FAK signaling pathway to stimulate cell viability and NO production, while also blocking further focal adhesion, estradiol-induced vascular endothelial injury, cell invasion, and migration [59].

Additionally, genistein hinders hypertension mainly caused by a high-salt diet. Severe oxidative damage and inflammation occur around the hypothalamic paraventricular nucleus due to a high-salt diet. In male Wistar rats on the high-salt model (8% NaCl), after being treated by genistein, their systolic pressure, diastolic pressure, and MAP showed various degrees of reduction, indicating the potential use of genistein in hypertension treatment. Based on the increments of glutathione disulfide (GSSH), SOD, Ac-FOXO1, GSH, SIRT1, Nrf2, HO-1, and NQO-1, genistein inhibited oxidative stress through the SIRT1/Nrf2 pathway, providing a basis for high-salt diet-induced hypertension treatment [60].

As a continuous pathophysiological procedure that is activated by a local redox imbalance to endothelial dysfunction, atherosclerosis (AS) is another serious CVD that can be alleviated by genistein [61]. Before treating AS, it is essential to understand the two hallmarks of AS: leukocyte adhesion and atherosclerotic plaques. Genistein alleviated the formation of these two AS factors to repair the cardiovascular system. It activated NO, boosting cyclic guanosine monophosphate and limiting leukocyte adhesion to the vessel wall [59]. Meanwhile, genistein suppressed platelet aggregation and adhesion to inhibit fibrous plaque formation, preventing the further development of AS.

In other words, genistein exhibits a multifaceted role in alleviating CVD by inhibiting inflammation, promoting the differentiation of abnormal cells, and reducing the NaCl content in the human body. This suggests a promising therapeutic approach for managing vascular inflammation and dysfunction caused by lifestyle and dietary factors. However, it is essential to conduct large-scale clinical trials to validate the efficacy and safety of genistein in the treatment of CVD and to investigate advanced drug morphological designs, such as nanoparticles or liposomes, which enhance absorption and tissue targeting. The role of genistein in treating PD, AD, DM, and CVD, as demonstrated in cell and animal studies, is summarized in Table 1.

### 4.5. Cancer

Cancer remains a leading cause of death worldwide, with a continued increase in incidence and complexity. Chemotherapy, radiation, immunotherapy, and surgery are common cancer treatments. However, adverse effects such as premature ovarian failure resulting from radiotherapy, adverse body reactions after surgical resection, and secondary poisoning infection based on drug accumulation evoke significant side effects in cancer treatment [7,62,63]. As an advanced cancer treatment technology, targeted therapy minimizes side effects and reduces the likelihood of drug resistance, thereby providing new options for patients [62,64,65].

Genistein regulates typical protein expressions to protect against acetaldehyde-mediated cell injury and other liver damage, as shown in Table 2. After being compared with five common flavonoids, genistein had a better ability to reduce TNF-α in the liver [66]. Genistein treatment significantly decreased alanine aminotransferase (ALT), aspartate aminotransferase (AST), TNF-α, Mcp-1, and CXCL1 levels in an alcohol-fed mouse model. HO-1, which repairs the dysfunction caused by acetaldehyde-induced hepatocellular carcinoma and reverses the rise in Nrf2, was stimulated by genistein and induced a decrease in Keap1 downstream, leading to the inhibition of liver cancer [67]. Moreover, hepatocellular carcinoma (HCC) is another common cause of death among patients with cirrhosis. An in vivo study found that after genistein treatment, the levels of MAD and hydrogen peroxide decreased, while the levels of GSH, SOD, and Nrf2 increased in the Sprague Dawley rat model. Compared with rats treated with 25 mg/kg genistein, the survival rate of the rats treated with 75 mg/kg genistein was three times higher, reflecting the dose-dependent property of genistein again. The significant decline in ALT, AST, GGT, alkaline phosphatase, and ERK-1 protein expression demonstrated that genistein was protective against liver damage. In addition, a decline in PDGF protein was observed only in the HCC control group, indicating that genistein restrains the further development of HCC-induced proteins and influences cell repair, which is affected by the activation of downstream signaling pathways, such as the MAPK and PI3K/Akt signaling pathways [68]. Furthermore, genistein downregulated lncRNA TTTY18 and reduced the number of SGK1, AktSer473, p38 MAPK, and Tyr323 pathways to inhibit the ulterior spread of colorectal cancer (CRC) [69].

Additionally, genistein is also widely used for DNA damage repair following ROS exposure [70,71]. Oxidative DNA damage contributes to DNA breakage and has long-term side effects [70]. Similarly, genistein activated the Nrf2/ARE pathway to reduce ROS generation and DNA injury in bronchial epithelial cells [7]. By targeting the DNA injury response and promoting G2/M cell cycle arrest, genistein increased the susceptibility of cancer cells to radiation, stimulated apoptosis, and inhibited the progression of cervical cancer [71].

Another important mechanism for genistein-targeted therapy is the regulation of the cell cycle and protein synthesis, which prevents the growth of malignant cells. An in vivo study in female Sprague Dawley rats demonstrated that genistein attenuated Bcl-2 expression and stimulated Bax expression, thereby increasing cytochrome C and caspase-3 expression. It upregulated ER-β and FOXL-2, downregulated TGF-β, reversed ovarian apoptosis, and blocked the growth of primordial follicles [62]. Correspondingly, genistein downregulated cyclin B1 and Bcl-2 by activating the NF-κB pathway at concentrations ranging from 5 to 20 μM. It inhibited HER-2 expression and phosphorylation, thereby restraining breast cancer development [72]. Genistein broke down cancer inhibitors of protein phosphatase 2A and increased the cytosolic Ca^2+^ buffering capacity of MCF-7 and T4D7 cells to decrease apoptosis and breast cancer cell proliferation [73]. It prevented the activation of G protein-coupled receptor-30 in breast cancer gene 1, causing phosphorylation of Akt and arresting the G2/M phase [74]. In CRC treatment, genistein similarly arrested the cell cycle at the G2/M phase to downregulate CDK4 and cyclin D1 expression in human salivary adenoid cystic carcinoma cell lines [63,75]. Thus, genistein inhibited the further spread of cancer cells by regulating specific proteins and controlling the cell cycle.

In summary, genistein offers promising therapeutic potential by mitigating the side effects of cancer treatment, exhibiting hepatoprotective properties, repairing oxidative DNA damage, and inhibiting the proliferation of malignant cells. Its mechanisms include protection against liver damage by regulating antioxidant pathways, repairing the DNA damage caused by ROS, and arresting the cell cycle to stunt tumor growth, highlighting its multifaceted role in targeted cancer therapy (Table 2).

## 5. The Role of Genistein in Recent Treatments

### 5.1. Pharmacokinetic Profile and Clinical Studies of Genistein

Genistein is present in plant raw materials as glycosides and is converted into an active aglycone for improved absorption by the human body after oral administration [76]. Viña and his team identified that the oral absorption of genistein is stable and results in minimal side effects in the body [36]. Both animal and human studies have demonstrated that the oral bioavailability of free genistein is low. For instance, its absolute bioavailability in rats is only 6.8%, and human pharmacokinetic studies have also reported low plasma and urinary levels of aglycone genistein. This poor oral bioavailability is largely due to the extensive metabolism of genistein in the body [77].

Based on this pharmacokinetic profile, several clinical studies have focused on the anti-inflammatory effects of genistein in preventing CVD, T2DM, and cancer (Table 3). For instance, genistein lowered the risk of hypertension-related complications (e.g., stroke and kidney disease), reduced heart strain, and promoted vascular health due to the decline in SBP, DBP, and MAP [78]. It also reduced the risk of DM in postmenopausal women [41]. Furthermore, in terms of blood sugar control, three months of genistein treatment lowered fasting blood sugar, A1C, and IR in postmenopausal women with DM, downregulated MDA levels, and stimulated thoracic aortic calcium levels to prevent further oxidative damage [42]. Kumar et al. studied the effects of genistein on prostate cancer (PCa), the most prevalent cancer among American men, across both African American men (AAM) and Caucasian men (CM). After a month of treatment, the PSA level in CM and the IGF-1/IGF-BP-3 ratio decreased, suggesting that genistein alleviated PCa to varying degrees in both groups. However, due to the limited number of participants with AAM, the effectiveness of genistein for PCa treatment in AAM requires further confirmation [79].

However, based on the lower rate of absorption of genistein, which accounts for only 20–40% of the gastrointestinal tract in enterohepatic cycling, the bioavailability of genistein is at a more fundamental level [80]. Thus, numerous researchers have expressed concerns about its future clinical application and sought alternatives to improve the bioavailability or efficacy of genistein.

### 5.2. Combination Therapy with Exercise or Existing Agents to Enhance Genistein’s Bioavailability and Efficacy

Some scholars have sought to improve the bioavailability and efficacy of genistein by combining it with exercise, existing drugs, or technologies. The synergistic effects of exercise and genistein have been studied in the context of retinal neovascularization, obesity induced by a high-fat, high-sugar (HFHS) diet, and non-alcoholic steatohepatitis (NASH). For instance, a previous study has suggested that retinal neovascularization, a visual disorder that occurs during the postmenopausal period or in patients with T2DM, can be treated with powerful antioxidant supplements or physical activity. Compared with the single application of either swimming or genistein, the co-treatment of genistein and swimming markedly reduces the levels of MDA, IL-6, and TNF-α, as well as the expression of vascular endothelial growth factor, in the retinal tissues of Wistar rats [81]. In addition, genistein and running were combined to mitigate the detrimental effects of an HFHS diet in male C57BL/6 mice with AD. The levels of pGSK-3β, p-IR, caspase 3, CP13, and APP in the combined treatment group were significantly lower than those in the treatment group, indicating a positive change in mice [82]. In previous postmenopausal animal models, exercise has been proven to have an estrogen-like effect in reducing fatty liver disease. Genistein relieved NASH primarily by exerting phytoestrogenic effects. F0 fibrosis observed in the combination treatment group demonstrated an improvement in liver fibrosis during NASH treatment, potentially halting further disease progression and promoting liver function recovery [83].

Likewise, combining genistein with metformin (MET) has been explored as a potential strategy to ameliorate non-alcoholic fatty liver disease (NAFLD). This combination aims to slow NAFLD progression by leveraging the ability of metformin to inhibit hepatic glucose production and increase insulin sensitivity in peripheral tissues, which is beneficial in the treatment of T2DM. Combining these two agents significantly decreased the amelioration of glucose tolerance, ALT, plasma TG, and liver TG in HFD-fed C57BL/6 mice compared to the groups treated with genistein and MET alone. Meanwhile, the combination of genistein and MET inhibited SREBP-1c expression induced by an HFD, a response that was not altered by the separate use of genistein or MET [84]. Additionally, some researchers have tried to supplement genistein with existing drugs for breast cancer treatment to identify their effects on cellular anticancer properties. The research team of Bezerra et al. reported that the combination of exemestane and genistein significantly reduced S-phase and cyclin E levels, enhanced the anti-proliferative and apoptotic effects, and altered the PARP/PARP ratio in MCF-7aro cells [85]. Another in vitro study on breast CSCs found that the combination of genistein and myokines was able to reduce colony and sphere formation in the MCF-7 cell model, highlighting the potential of these treatments to inhibit the self-renewal and stemness of CSCs. Additionally, the expressions of SOX2 and OCT4 (both were CSC markers) were suppressed after the co-treatment, which was more effective than the individual treatments [86].

As a promising approach to enhance cancer treatment outcomes, neutron radiotherapy addresses the limitations of monotherapy, minimizes the risk of drug resistance, and improves patient prognosis. Interestingly, Khamesi and his team discovered that the combination of neutron radiotherapy and genistein, although considered a radiation sensitizer, did not exhibit a better efficacy in killing prostate cancer cells than neutron radiation and genistein alone [87]. Although specific combination therapies with genistein have shown excellent synergistic effects and improved therapeutic outcomes in some cases (Table 4), it is undeniable that not all combinations guarantee superior efficacy, as some may offer no additional benefit compared to monotherapies.

### 5.3. Advances in the Use of Nanotechnology for Genistein Delivery to Enhance Bioavailability

Nanotechnology has emerged as a transformative technique in medical research, providing innovative solutions for drug development and improvement [88]. Its advantages, such as enabling the precise targeting of therapeutic agents, facilitating the delivery of large biomolecules, and improving the water solubility and bioavailability of drugs, have attracted extensive research interest from scientists [88,89]. They have developed numerous nanoscale structures, including liposomes, polymer-based nanoparticles, micelles, dendrimers, solid lipid nanoparticles, nanoemulsions, nanocapsules, ceramic particles, and metallic nanoparticles, to further enhance the effectiveness of their drug delivery systems [89]. In recent literature reviews, Khan et al. and Syahputra et al. have highlighted the potential effects of flavonoid nano-formulations (quercetin in eudragit-coated liposomes, red ginger flavonoids encapsulated in nanoemulsion systems, EGCG formulated in PLA-PEG (polylactic acid–polyethylene glycol) nanoparticles, etc.) in inhibiting ROS formation, reducing systolic blood pressure, and preventing or treating cancer [89,90]. Similarly, nanotechnology has significantly advanced the formulation and delivery system of genistein, utilizing lipid nanoparticles, liposomes, tocotrienol-rich nanoemulsions, polymeric nanoparticles, dextran complexes, chitosan complexes, and Fe_3_O_4_ nanoparticles with carboxymethylated chitosan. These methods enable precise control over drug release and enhance the solubility and stability of genistein [91]. Recently, the novel genistein-loaded solid lipid nanoparticles (SLNs) developed by Obinu et al. have demonstrated excellent bioavailability, stability, and a high drug loading capacity. In their studies, SLNs could be absorbed by the intestinal mucosa and Caco-2 cells, indicating that improved intestinal lymphatic absorption may be achieved by minimizing the first-pass metabolic effect of genistein [92]. Moreover, the development of genistein-loaded nanoparticles not only enhances these physicochemical and pharmacokinetic properties but also enables more precise targeting of specific cells, thereby decreasing the risk of adverse effects on human primary cells or healthy cells. For instance, the genistein–gold nanoparticle conjugates Gen@AuNPs1 and Gen@AuNPs2 developed by Vodnik et al. have been reported to not only effectively suppress cancer cell growth but also enhance the targeting of cancerous phenotypes [93]. In summary, the integration of nanotechnology with genistein holds great promise for overcoming traditional pharmacological limitations, enhancing therapeutic efficacy, and is essential for more precise, safer, and effective treatments.

### 5.4. Therapeutic Doses of Genistein by Disease Entity

As mentioned above, genistein exhibits dose-dependent, dual effects on regulating oxidative stress. At relatively low concentrations, it acts as an antioxidant by upregulating detoxification and antioxidant enzymes through various signaling pathways, including the PI3K/Akt, NF-κB, and Nrf2/HO-1 pathways, thereby reducing oxidative stress, inflammation, and neuronal toxicity, which highlights its therapeutic potential for brain injury and neurodegenerative diseases. However, exposure to high-dose genistein has a pro-oxidative role, promoting the production of ROS and leading to increased oxidative damage and inflammation. This heightened oxidative stress can decrease the viability of cancer cells, but it also raises the risk of hormonal imbalances and hormone-sensitive diseases due to its phytoestrogenic properties. Overall, genistein also appears to elicit varying responses depending on the treatment concentrations. Thus, this section summarizes the therapeutic doses of genistein reported in recent studies, categorized by different disease entities, to provide practical information for clinical reference and the development of functional foods.

According to the meta-analysis provided by Lei et al. and numerous experimental studies, it is clear that genistein treatment in the AD rat model (treatment concentration: 0.022 to 150 mg/kg b.w./day; time duration: from 6 days to 9 months) led to different outcomes [94]. Moreover, the National Institutes of Health (NIH) in the United States recommends that the daily dietary intake of isoflavones for adults should be in the range of 30 to 50 mg [6]. Certain clinical studies have demonstrated that taking 60 mg of genistein orally twice a day for 12 months significantly enhances cognitive performance in AD patients and exhibits a promising effect by decreasing Aβ plaques within the anterior cingulate gyrus [95]. Unfortunately, this dosage in the trial is much higher than the daily intake recommended by the NIH; as a result, the appropriate dosage for individuals with AD still needs to be established. Unlike the preclinical studies in the AD model, almost all of the genistein experimental groups in the LPS-induced inflammatory PD rat model or the 6-OHDA-induced PD rat model received genistein via i.c.v. injection or i.p injection [26,96,97]. For example, a single, high-dose intraperitoneal injection of genistein (10 mg/kg b.w.) ameliorated rotational behavior and protected neurons in the substantia nigra pars compacta (SNC) in 6-OHDA-induced rats [96,97]. However, there is currently a lack of clinical data in patients with PD, and further research is required to determine the effective dose in humans.

A randomized, double-blind, placebo-controlled clinical trial in postmenopausal women with T2DM has commonly used genistein at a dose of 108 mg/day for 12 weeks, resulting in improved glycemic control and reduced serum lipid levels [42]. Another clinical study investigating the effects of genistein on insulin resistance in pre-diabetic individuals suggested that a genistein supplement (50 mg/day) for 2 months significantly attenuated insulin resistance, which in turn prevented metabolic abnormalities and further progression to diabetes [45]. Thus, in human-based research, the doses of genistein ranging from 50 to 108 mg/day for 2 to 3 months effectively improved glycemic regulation, lipid control, and insulin sensitivity in DM patients, leading to effective DM treatment. Generally, in vivo studies administered a higher level of genistein to determine its anti-DM and antioxidative properties. Gilbert et al. and Yang et al. have mentioned that genistein at 8 mg/kg b.w., the average daily consumption from a soy-rich human diet, exhibited a limited effect on diabetic rats, whereas genistein at 30 mg/kg significantly improved glucose metabolism [98,99]. In summary, both clinical and animal studies suggest that genistein has huge potential to regulate blood glucose and improve metabolic profiles within a specific dosage range; however, the effective dosage in animal experiments is significantly higher than that in the human diet and should be taken into consideration.

Regarding the dose of genistein in cancer treatment, the optimal dose differs depending on the type of cancer and the specific study parameters. In an azoxymethane-induced colon cancer male Sprague Dawley rat model, the genistein intervention (140 mg/kg b.w.) for 13 weeks was reported to prevent early colon neoplasia [100]. In an animal model of liver cancer, the administration of genistein for 15 days (15 mg/kg b.w.) effectively treated liver cancer by inducing apoptosis in HCC rats [101]. Similarly, genistein treatment (75 mg/kg b.w.) for 16 weeks enhances liver function in HCC rats [68]. In a study on ovarian cancer, the administration of genistein (5 mg/kg body weight, administered intraperitoneally for 7 days) showed positive effects on antioxidant activity and ovarian tissue protection [62]. However, it is notable that the impact of exposure to genistein on women with estrogen-dependent breast cancer remains controversial [102]. In a mouse model designed to replicate the low-estrogen environment of postmenopausal women, genistein treatment may present adverse health effects, including promoting estrogen-dependent tumor growth. Thus, the optimal therapeutic dose of genistein should be tailored to the specific disease, patient population, and treatment goals. Its safety and potential adverse effects should be considered carefully. Further animal and clinical trials are needed to determine the recommended dosage for each disease entity.

## 6. Conclusions

Oxidative stress induces plaque aggregation, insulin signaling, DNA damage, and endothelial dysfunction, which further induce or exacerbate various diseases, including neurodegenerative diseases, metabolic diseases, and cancer. This review systematically elucidates the detailed molecular mechanisms, particularly the roles of the Nrf2/HO-1, PI3K/Akt, and NF-κB pathways, clarifying how genistein exerts its antioxidant, anti-inflammatory, and anti-apoptotic effects in five common diseases (PD, AD, DM, CVD, and cancer) that are prevalent in the population. As a plant-derived compound, genistein is recognized as a promising therapeutic option for symptom relief due to its abundant natural availability, dietary applicability, and favorable side effect profile. In conclusion, a rich body of evidence suggests that it alleviates disease progression by inhibiting oxidative stress, inflammation, apoptosis, and endothelial dysfunction, all of which are closely related to oxidative stress. To improve its bioavailability and therapeutic efficacy, genistein has been combined with exercise, existing drugs, and advanced delivery systems such as nanoparticles, enhancing oral absorption. However, the optimal therapeutic dose of genistein for specific diseases, as well as the efficacy and synergistic effects of some combined therapies, remain obstacles to the use of genistein in disease prevention and the development of functional foods. Further animal and clinical trials, as well as systematic literature reviews, are needed to explore its efficacy and bioavailability.

## Figures and Tables

**Table 1:** A summary of preclinical studies of genistein in treating Parkinson’s disease (PD), Alzheimer’s disease (AD), diabetes mellitus (DM), and cardiovascular disease (CVD).

Targeting Diseases	Experimental Model	Treatment	Outcome Characteristics	References
PD	Human SH-SY5Y cells overexpressing the A53T mutant of α-synuclein	Incubated with 20 μM genistein and rotenone for 24 h.	Rotenone-induced cell death, mitochondrial oxidative stress, and apoptosis ↓ Protein expression level of nuclear NFE2L2, HMOX1, and p-Akt ↑	[27]
	Lipopolysaccharide (LPS)-induced ovariectomized rats	Orally gavage with genistein (10 mg/kg) once daily for 14 consecutive days. Ovariectomy (OVX) was performed to eliminate the endogenous estrogen effect.	Apomorphine-induced rotational behavior ↓ Proinflammatory factor level (TNF-α and IL-1β) ↓ Protein expressions of COX-2 and iNOS ↓ Suppressing LPS-induced activation of MAPK and IκB signaling pathways through GPER and IGF-1R	[26]
	Transgenic *Drosophila* expressing normal human αS panneurally	Treated with 0–40 μM genistein.	Lifespan ↑ Inhibition of oxidative stress damage: GSH ↑ GST activity ↓ LPO level ↓ PC content ↓ Dopamine content ↑ Monoamine oxidase activity ↓	[28]
AD	Amyloid beta (Aβ)_25–35_-treated SH-SY5Y cells	After being treated with 0–50 μM genistein for 90 min, cells were exposed to Aβ_25–35_ at a concentration of 20 mM for 24 h.	Aβ-induced cell death ↓ Protein and mRNA expression of *HO-1* ↑ Activation of Nrf2//HO-1/PI3K signaling	[33]
	Aβ_25–35_-treated Rat primary hippocampal neurons	After incubating with 0–1 μg/mL genistein for 24 h, neurons were continuously exposed to Aβ_25–35_ for 3 days.	Aβ-induced cytotoxicity and necroptosis ↓ Aβ-induced LDH release, ROS accumulation, and MDA production ↓ Activating PI3K/Akt phosphorylation via α7nAChR signaling Activation of endogenous Nrf2/Keap1 transcription factors ↑	[35]
	Streptozotocin (STZ)-induced male Wistar rat model of the sporadic form of AD	Rats were administered 150 mg/kg b.w. via an orogastric probe once a day for 30 or 90 days.	Locomotor activity, memory and cognitive ability ↑ Protein expression level of levels of APP, total Aβ, Aβ_40_, Aβ_42_, and p-tau in the cortex, hippocampus, and the rest of the brain ↓ Autophagy ↑	[38]
DM	Methylglyoxal (MG)-treated *EA.HY926* human endothelial cells	Cells were pre-incubated for 2 h with genistein (0–100 µM) before co-treatment with 250 μM MG for 24 h.	MG-induced toxicity and ROS formation ↓ G0/G1 percentage ↑ Prevented MG-induced apoptosis via Nrf2 activation and MAPK-mediated signaling pathway regulation.	[50]
	Ovariectomized diabetic rats	Animals underwent swimming training (1 h/day) or received genistein (35 mg/kg b.w.) or a combination of both for eight weeks.	Inflammatory protein levels of IL-1β, Nf-κB, and TNF-α ↓ Anti-inflammatory protein levels of SIRT1 ↑	[41]
CVD	High-fat-diet (HFD)- and LPS-induced chronic vascular inflammation in C57BL/6 mice	Intraperitoneal injection of LPS, combined with a HFD, was used to create the chronic vascular inflammation model. Administered orally with genistein (10 mg/kg b.w.) for 20 weeks.	Expression of inflammation-associated factors (mRNA expression of *TNF-α* and *IL-6*, as well as iNOS and NF-κB p65 protein levels) ↓ miR-21 ↓	[57]
	Δ^9^-THC-induced C57BL/6J male mice	The mice were randomized into three groups: control group; Δ^9^-THC treated group (1 mg/kg b.w./day); Δ^9^-THC (1 mg/kg b.w./day)- and genistein (50 mg/kg b.w./day)-treated group.	Reversed Δ^9^-THC-induced endothelial dysfunction, oxidative stress, and inflammation NF-κB phosphorylation ↓	[58]
	Estradiol-induced human umbilical vein endothelial cells (HUVECs)	Cells were treated with or without genistein in the presence of estradiol.	Cell viability ↑ NO level ↑ ROS formation ↓ Cell invasion and migration ↓ FAK protein expression ↓	[59]

Notes: NFE2L2, nuclear factor erythroid 2-related factor 2; HMOX1, heme Oxygenase 1; TNF-α, tumor necrosis factor alpha; IL-1β, interleukin-1 beta; IκB, inhibitor of NF-κB; COX-2, cyclooxygenase-2; iNOS, inducible nitric oxide synthase; GPER, G protein-coupled estrogen receptor; IGF-1R, insulin-like growth factor 1 receptor; MAPK, mitogen-activated protein kinase; GSH, glutathione; GST, glutathione S-transferase; LPO, lipid peroxidation; PC, protein carbonyl; Nrf2, nuclear factor erythroid 2-related factor 2; Nf-κB, nuclear factor kappa B; Keap1, Kelch-like ECH-associated protein 1; APP, amyloid precursor protein; ROS, reactive oxygen species; MDA, malondialdehyde; NO, nitric oxide; FAK, focal adhesion kinase; ↓, decrease; ↑, increase.

**Table 2:** A summary of preclinical studies of genistein in treating cancer.

Targeting Diseases	Experimental Model	Treatment	Outcome Characteristics	References
Liver cancer	Alcohol-fed male ICR mice	Mice in the flavonoid groups were orally administered five different kinds of flavonoids, respectively (quercetin, apigenin, naringenin, epigallocatechin gallate, and genistein; 0.3 mmol/kg b.w.), 1 h before the alcohol consumption for 5 weeks.	Hepatic function ↑ Prevented dyslipidemia Hepatic lipid peroxidation and oxidative stress ↓ Hepatic inflammatory stress ↓ Hepatic fibrosis and apoptosis ↓	[66]
	Chronic alcohol-fed mice	Mice were subjected to a Lieber–DeCarli alcohol liquid diet with or without genistein (1 mg/kg b.w./day mixed into their diet) for 8 weeks.	Liver injury and hepatic steatosis ↓ Hepatic inflammatory cell infiltration ↓ Ameliorated alcohol-induced hepatic oxidative stress, ER stress, and mitochondrial dysfunction Acetaldehyde-induced hepatocyte apoptosis ↓ HO-1 restoration and upregulation of NRF2 are involved in the preventive effect of genistein against ALD	[67]
	Thioacetamide (TAA)-induced Hepatocellular carcinoma (HCC) in Sprague Dawley rats	Rats were randomly divided into five groups: control group; genistein-treated group (75 mg/kg b.w., orally intake); HCC group (200 mg/kg b.w. TAA, i.p., twice a week); HCC + low dosage of genistein-treated group (25 mg/kg b.w., orally intake); and (v) HCC + high dosage of genistein-treated group (75 mg/kg b.w., orally intake). Treatments lasted for 16 weeks.	HCC-induced oxidative stress ↓ (hepatic MDA, and hydrogen peroxide ↓; hepatic Nrf2, GSH, and SOD levels ↑) Liver function ↑ (ALT, AST, alkaline phosphatase, and GGT serum levels ↓; serum albumin levels ↑) Protein expression of PDGF ↑, versican ↑, PKC ↓, and ERK-1 ↓	[68]
Ovarian cancer	γ-radiation to induce POF in female Sprague Dawley rats	Rats were administered a single intraperitoneal injection of genistein (5 mg/kg body weight) for 7 days, followed by exposure to a 3.2 Gy single dose of γ-rays on the 7th day.	Protected the ovarian tissue from hemorrhage and fibrosis Oxidative stress ↓ (GSH level and GPx activity ↑) mRNA expression of *Bax* ↓ and *Bcl-2* ↑ Optical densities of Cytochrome c and Caspase 3 ↓ Ovarian mRNA expression of *ER-β* ↑, *FOXL2* ↑, and *TGF-β* ↓	[62]
Colorectal cancer	Human malignant cell line of SW480	Cells were treated with different concentrations of genistein (0, 25, 50, and 100 μM) for 48 h.	Cell viability ↓ Cell apoptosis ↑ Cellular migration ↓ Protein expression of TTTY18, SGK1, Akt^Ser473^, p38 MAPK^Tyr323^ ↓	[69]
	Tumor-bearing nude mice	The mice were treated with 0, 20, 30, and 60 mg/kg b.w. genistein for 14 consecutive days.	Body mass ↓ Tumorous TGF-β1 and TTTY18 ↓ Intracellular numbers of SGK1, Akt^Ser473^, p38 MAPK^Tyr323^ positive cells ↓	[69]

Note: ALT, alanine transaminase; AST, aspartate transferase; GGT, gamma-glutamyl transferase; ER-β, estrogen receptor beta; Bax, Bcl-2-associated X protein; Bcl-2, β-cell lymphoma 2; FOXL2, forkhead box protein L2; TGF-β, transforming growth factor beta; SGK, serum and glucocorticoid-inducible kinase; ↓, decrease; ↑, increase.

**Table 3:** Therapeutic effects of genistein in clinical trials.

Disease	Experiment Model	Treatment	Outcome Characteristics	References
AD	24 prodromal AD patients (54 to 76 years old) in a double-blind, placebo-controlled, and bicentric clinical trial	Randomly received genistein (one capsule/time, 60 mg/capsule) or placebo orally twice per day for up to 12 months. Orally twice per day for up to 12 months	Amyloid-beta deposition uptake in the anterior cingulate gyrus ↓ Individual cognitive behavior ↑ No reported deaths or serious adverse events	[36]
DM	58 postmenopausal women with T2DM (randomized, double-blind, placebo-controlled clinical trial)	Randomly received genistein (two capsules/day, 54 mg/capsule) or placebo orally for 12 weeks.	FBS, A1C, TG, and MDA ↓ TAC, HDL-C, and QUICKI ↑	[42]
Prostate cancer	70 participants, of whom 36 participants (25 CM, 6AAM) were randomized to the isoflavone group and 34 (25 CM, 7AAM) to the placebo group	Administered isoflavones (20 mg BID) or placebo for 3–6 weeks.	No changes in serum steroid hormones PSA levels in CM ↓ IGF-1/IGF-BP-3 ↓ Ki-67 expression in the placebo group ↑	[79]

Note: FBS, fasting blood glucose; A1C, glycated hemoglobin; TG, triglyceride; MDA, malondialdehyde; TAC, total antioxidant capacity; HDL-C, high-density lipoprotein cholesterol; QUICKI, quantitative insulin sensitivity check index; AAM, African American men; CM, Caucasian men; PSA, prostate-specific antigen; ↓, decrease; ↑, increase.

**Table 4:** Synergistic therapeutic effects of genistein combined with exercise or conventional drugs in preclinical models.

Disease	Experiment Model	Treatment	Outcome Characteristics	References
Obesity and AD	High-fat and high-sugar (HFHS) mouse model of neurodegeneration	Mice were treated with genistein (600 mg/kg in HFHS diet), exercise training, or a combination of both for 12 weeks.	Body weight, adipose mass, and inflammatory marker TNF-α ↓ Key proteins’ expression involved in AD (pGSK-3β/GSK, Aβ, ADAM10, Caspase-3, pIR/IR, p-IRS/IRS) ↓	[82]
Non-alcoholic steatohepatitis (NASH)	NASH model of OVX rats fed with high-fat high-fructose (HFHF) diet	Rats were given genistein (16 mg/kg b.w.), engaged in moderate running exercises, or both for 5 weeks.	Did not provide additional benefits for NASH in OVX rats fed with HFHF diet.	[83]
Non-alcoholic fatty liver disease (NAFLD)	HFD-fed mice model of NAFLD	Mice were administered 0.23% metformin (MET, 2.3 g/kg diet) combined with 0.2% genistein (2 g/kg diet), or MET and genistein alone.	Body and liver weights ↓ FBG, fasting plasma insulin, HOMA-IR, glucose tolerance, ALT, AST, plasma TG, and liver TG ↓ Steatosis ↓ Gene expression of *FAS*, pro-inflammatory (*TNFα*, *IL-1β*, and *IL-6*), *PEPCK*, and *G6Pase* ↓ Protein expression of pGSK-3β ↑ Better efficacy than treatment with genistein or metformin alone	[84]
Breast cancer	ER^+^ aromatase-overexpressing human breast cancer cell line MCF-7aro	Cells were treated with genistein (0.5–25 µM), with or without exemestane (Exe), anastrozole (Ana), or letrozole (Let ) (1, 5, and 10 µM) for 3 days.	Combination of genistein with Ana or Let negatively impacts the therapeutic efficacy of aromatase inhibitors. Genistein enhanced the anticancer properties of Exe. Hormone targets are not affected by this combination treatment.	[85]
	Human breast cancer MCF-7 cells	Cells were treated with various concentrations of myokines, genistein, or their combination for 72 h.	A larger reduction in colony formation than myokines or genistein alone. Higher reduction in sphere formation Higher decrease in *SOX2* and *OCT4* gene expressions	[86]

Note: pGSK-3β, brain phosphorylated glycogen synthase kinase; IR, insulin receptor; IRS, insulin receptor substrate; HOMA-IR, homeostasis model assessment of insulin resistance; ALT, alanine transaminase; AST, aspartate transaminase; TG, triglyceride; TNFα, Tumor necrosis factor alpha; IL-1β, Interleukin-1 beta; IL-6, Interleukin-6; ↓, decrease; ↑, increase.

## Abbreviations

AAM: African American men; AD: Alzheimer’s disease; ALT: alanine aminotransferase; AMPK: adenosine 5′-monophosphate-activated protein kinase; APP: amyloid precursor protein; AS: atherosclerotic; AST: aspartate aminotransferase; Bax: Bcl-2-associated X protein; Bcl-2: β-cell lymphoma 2; Bid: Bcl-2-associated X protein; CAT: catalase; CM: Caucasian men; COX-2: cyclooxygenase-2; CRC: colorectal cancer; CVD: cardiovascular disease; DA: dopamine; DM: diabetes mellitus; DOPAC: 3,4-Dihydroxyphenylacetic acid; ERK: extracellular signal-regulated kinase; GPx: glutathione peroxidase; GSH: glutathione; GSSH: glutathione disulfide; HCC: hepatocellular carcinoma; HDAC3: histone deacetylase 3; HER: human epidermal growth factor receptor 2; HFD: high-fat diet; HFHS: high-fat high-sugar; HIBD: hypoxic-ischemic brain damage; HO-1: hemoxygenase 1; HOMA-IR: homeostatic model assessment of insulin resistance; HVA: homovanillic acid; IGF-1R: insulin-like growth factor 1 receptor; IL-1β: interleukin-1β; IL-6: interleukin-6; iNOS: inducible nitric oxide synthase; IR: insulin resistance; IκB: inhibitor of NF-κB; JNK: jun N-terminal kinase; Keap1: Kelch-like ECH-associated protein 1; MAD: multiple ascending dose; MAPK: mitogen-activated protein kinase; mCRC: metastatic colorectal cancer; MET: metformin; MG: methylglyoxal; NAFLD: non-alcoholic fatty liver disease; NAMPT: nicotinamide phosphoribosyltransferase; NASH: non-alcoholic steatohepatitis; NF-κB: nuclear factor κ Β; NNK: 4-(methylnitrosamino)-1-(3-pyridyl)-1-butanone; NIH: National Institutes of Health; Nrf2: nuclear factor erythroid 2-related factor 2; PCa: prostate cancer; PD: Parkinson’s disease; PHB1: prohibitin 1; PI3K/Akt: phosphatidylinositol 3-kinase/protein kinase 3; RhoA: Ras homolog family member A; RNS: reactive nitrogen species; ROS: reactive oxygen species; SIRI1: Sirtuin 1; SN: substantia nigra; SOD: superoxide dismutase; T2DM: type 2 diabetes mellitus; TG: triglyceride; TLR4: toll-like receptor 4; TNF: tumor necrosis factor; TNF-α: tumour necrosis factor-α; VECs: vascular endothelial cells;; αS: α-synuclein; 6-OHDA: 6-hydroxydopamine; SLNs: solid lipid nanoparticles; NAD^+^, nicotinamide adenine dinucleotide, oxidized form.
